# Autism Spectrum Disorder and collective catering service: results of the pilot study FOOD-AUT

**DOI:** 10.3389/fnut.2023.1298469

**Published:** 2024-01-10

**Authors:** Maria Vittoria Conti, Sara Santero, Chiara Breda, Sara Basilico, Giorgia de Filippo, Alessia Luzzi, Luana Voto, Rebecca Cavagnola, Chiara Elena Tomasinelli, Hellas Cena

**Affiliations:** ^1^Laboratory of Dietetics and Clinical Nutrition, Department of Public Health, Experimental and Forensic Medicine, University of Pavia, Pavia, Italy; ^2^School of Nutrition Science, University of Milan, Milan, Italy; ^3^Department of Translational Medical Science, University of Naples Federico II, Naples, Italy; ^4^Department of Brain and Behavioral Sciences, University of Pavia, Pavia, Italy; ^5^Clinical Nutrition Unit, General Medicine, ICS MAUGERI IRCCS, Pavia, Italy

**Keywords:** Autism Spectrum Disorder, food selectivity, collective catering, menus, nutrition, human health

## Abstract

**Objective:**

Individuals with Autism Spectrum Disorder (ASD) often exhibit a low dietary diversity due to food selectivity that leads them to a marked preference for high-energy-density food, exposing them to risk of malnutrition. Despite these aspects, specific recommendations and targeted menus for this population are missing. The pilot study FOOD-AUT addresses this issue by developing canteen menus meeting the nutritional and sensory needs of adults with ASD, aiming to reduce their food selectivity, and consequently improving their health.

**Methods:**

The project, funded by Gruppo Pellegrini S.p.A, was conducted at the daycare service of Sacra Famiglia Onlus Foundation, between March-2022 to March-2023. The study was divided into two phases. Observational phase: a comparison was made between the enrolled subjects’ nutritional needs and the nutrient content of the administered menus during the daycare service. Then mealtime compliance was assessed using standardized meal evaluation forms, both quantitative and qualitative. Intervention phase: canteen menus targeted to the individuals’ nutritional and sensory needs were administered and their acceptability was evaluated.

**Results:**

Twenty-two individuals with ASD, aged 19–48, 72.7% males, were enrolled. Overweight and obesity prevalence were 54.5 and 18.2%, respectively. The observational phase showed how the most accepted foods had specific sensorial characteristics in line with the scientific literature. Adapting the menus improved food acceptance and reduced food waste.

**Conclusion:**

The results highlighted the need for adapted menus and greater attention to the way meals are delivered and consumed to improve nutritional status and therefore health of this population at increased risk of malnutrition.

**Clinical trial registration:**

ClinicalTrial.gov, unique identifier: NCT05978895.

## 1 Introduction

Autism Spectrum Disorder (ASD) is a heterogeneous set of broad-spectrum neurodevelopmental disorders that includes impairments in social interaction, language, communication, and imaginative play (1). It also includes restricted, repetitive, and stereotyped patterns of behavior, activities, and interests (1). According to the latest revision of the Diagnostic and Statistical Manual of Mental Disorders (DSM-5), the severity of ASD and support required, which describes the degree of impairment of the individual with ASD, can be divided into three different levels from “support needed” (level 1) to “very significant support required” (level 3) (1). In the last two decades, the prevalence of ASD globally has undergone a significant increase, linked both to changes in diagnostic criteria and to greater scientific evidence and increased knowledge of the disorder by the general population (2). In Italy a prevalence of 1 child out of 77 (ages 7–9 years) has been estimated (3). Furthermore, about 75% of patients with ASD have comorbidities such as psychiatric or neurological disorders that complicate the psycho-physical picture (2). This population also shows a significantly higher prevalence of overweight, obesity and constipation than the neurotypical population (4). Studies reported a higher prevalence of overweight (19% compared to 16%) and obesity (30.4% compared with 23.6%) between individuals with ASD and neurotypical ones (5).

Thus, in the last few years, the scientific community has investigated the relationship with food in ASD individuals, highlighting the presence of food selectivity, behavioral rigidity, specific meal rituals, and neophobia, behaviors that lead to very restricted food choices and consequently the elimination of entire food groups (6, 7). In detail, people with ASD tend to prefer foods characterized by soft or semi-liquid consistencies, low-intensity colors, and delicate tastes, not appreciating strong smells and high temperatures (6, 7). They also show a marked preference for ultra-processed foods, characterized by high energy density, high content of simple sugars, saturated fats, salt, and with a low minerals and vitamins content, as well as for food products of specific brands (6, 7). Given their preferences, individuals with ASD frequently follow an unhealthy diet with low dietary diversity, exposing them to a greater risk of developing metabolic disorders and different kinds of malnutrition conditions such as overweight or obesity and/or micronutrient deficiencies, resulting in further health deterioration (8). Moreover, ASD population is more exposed to gastrointestinal (GI) symptoms (9) which are four times more prevalent compared to the neurotypical population (10). Among GI symptoms, diarrhea and constipation are the most reported (11) and they may be associated both with an altered gut microbiota and food selectivity. Specifically, the latter attitude can impact negatively on intestinal microbiota balance leading to dysbiosis due to inadequate intakes of protein, dietary fiber, and essential fatty acids (9).

In Italy, 78,242 individuals with ASD are taken in charge by center-based services, which play a crucial role in their daily management (12). In this adapted setting, where the mealtime is managed by collective catering services, the absence of nutritional recommendations and menus targeted to this population is registered, determining a higher risk of food rejection. In addition, in order to improve meal acceptance, it is necessary to consider the environment in which the meal takes place, as well as the plating and service.

In this complex scenario, the FOOD-AUT pilot study aims to positively impact the health condition of adults with ASD, improving food acceptability and increasing dietary diversity through the development of menus aimed at collective catering service adapted to the nutritional and sensory needs of this population.

## 2 Methods

The present pilot study was conducted at the daycare service of Sacra Famiglia Onlus Foundation in Cesano Boscone, Italy, between March 2022 and March 2023. Approval for the study was obtained from the competent Ethics Committee of the University of Pavia (Comitato Etico del Dipartimento di Scienze del Sistema Nervoso e del Comportamento – sez. di Psicologia), project. n. 114/22. The study was performed in accordance with the principles of the Helsinki Declaration and was in line with the terms of Good Clinical Practice. The study was registered on clinicaltrial.gov with the following unique identifier: NCT05978895.

The inclusion criteria were the following: diagnosis of ASD (according to DSM-5 diagnostic criteria) including all the three severity levels (1); being a patient of Sacra Famiglia Onlus Foundation; aged ≥18 years; written informed consent signed. For each respondent the guardian parents signed written informed consent.

The study was divided into two main phases: Work Package (WP) 1 and 2.

### 2.1 Work package 1: observational phase

This first phase consisted in several activities aimed at evaluating the nutritional adequacy and sensory acceptance of canteen menus aimed at adults with ASD and identifying any critical issues associated with mealtime.

#### 2.1.1 Analysis of menus administered at the Sacra Famiglia Onlus Foundation

All the menus administered by Pellegrini S.p.A. at the daycare service were analyzed to assess the bromatological content of the foods (i.e., energy, macro- and micronutrients composition) using the Food Composition Database for Epidemiological Studies in Italy (BDA) (13). The results were compared with the nutritional needs of the enrolled subjects according to their age, sex, anthropometric measurements (14).

#### 2.1.2 Mealtime compliance assessment

**Figure 1:**
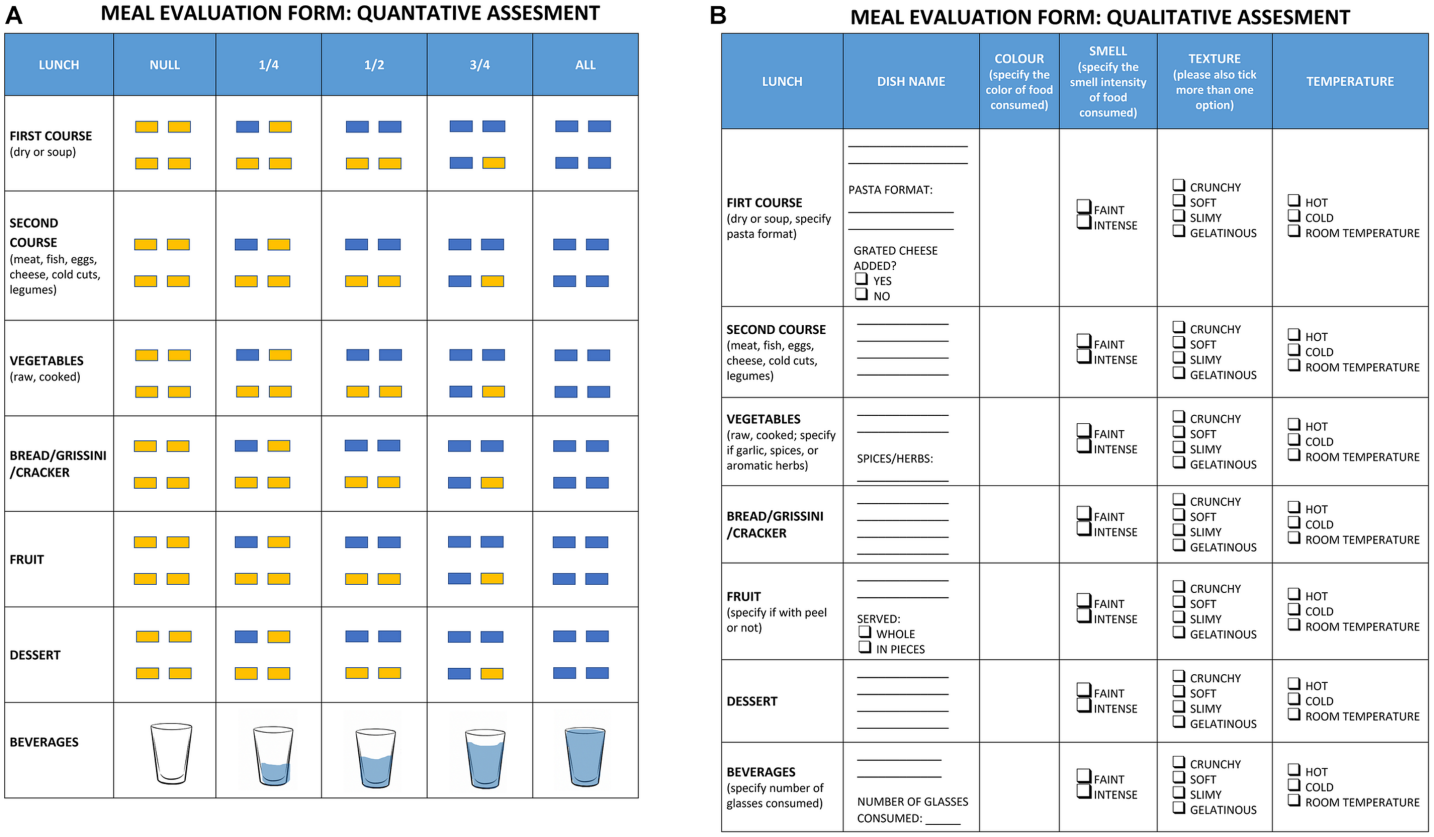
Meal evaluation forms. Shows the meal evaluation forms used for quantitative assessment **(A)** and qualitative assessment **(B)**.

Meal evaluation forms, a standardized tool (15) with the purpose of monitoring and evaluating actual meal consumption, were used to assess the amount of meal intake (Figure 1A).

The quantitative evaluation form had a section for each type of meal consumed: first course, second course, vegetable side dish, bread/grissini/cracker, fruit/yogurt, and beverages. For each of these categories, the box corresponding to the quantity of the dish consumed was checked. To ease compilation, the quantity consumed was expressed both numerically (null, ¼, ½, ¾ and all the plate) and graphically by means of four rectangles (representing a dish divided into four sections), which, respectively, changed from yellow (not consumed) to blue (consumed). For beverages, a stylized glass with a gradual filling of water was preferred as a graphical representation.

For the quantitative evaluation of food consumption, a score was assigned to the four possible amounts of dish consumed: zero consumption (score = 0); consumption of ¼ of the plate (score = 0.25); consumption of ½ of the plate (score = 0.5); consumption of ¾ of the plate (score = 0.75) and consumption of the whole dish (score = 1).

In addition to the quantitative assessment, a qualitative one followed, aimed at identifying the degree of acceptance with respect to the sensory characteristics of the food consumed (Figure 1B).

For each of the above categories, the qualitative evaluation form included the exact name of the dish (e.g., risotto or tomato pasta), its color (one or more), smell intensity (faint or intense), texture (crunchy and/or soft and/or slimy and/or gelatinous), and temperature (hot, cold, room temperature). For some dishes there was a request for more details. For pasta dishes whether the pasta format was short or long and whether there was the addition of grated cheese. For vegetables whether it was raw or cooked and whether it was served with garlic, spices, or aromatic herbs. For fruit whether it was served in pieces or whole (with peel or without peel). For beverages, it was also asked to specify the number of glasses consumed.

For each subject enrolled, a total of 12 meal evaluation forms were collected on three non-consecutive weekly days (for a total of 4 weeks). As the menus administered by Pellegrini S.p.A. covered 4 weeks, consequently over the 12-week survey period, the menu was repeated 3 times. Both the qualitative and quantitative forms were filled out by qualified operators of the Sacra Famiglia Onlus Foundation, who assist the individuals daily during lunches eaten at the facility. During the meals, the enrolled subjects were divided into tables of small groups of 4 or 5 people, and each group was supervised by a single trained operator who oversaw filling out the quantitative and qualitative forms. Throughout the project, each group was always followed by the same operator. Prior to data collection, the research team of the Laboratory of Laboratory of Dietetics and Clinical Nutrition, trained the operators on how to correctly fill out the forms.

### 2.2 Work package 2: interventional phase

This phase consisted of administering canteen menus adapted according to the nutritional and sensory needs of individuals with ASD and assessing the degree of food acceptance by the enrolled subjects in WP1.

For the evaluation of meal acceptance, what was performed for WP1 was repeated and a total of 12 meal evaluation forms for participants were collected on three non-consecutive weekly days (for a total of 4 weeks).

### 2.3 Statistics

Analyses were conducted with Rstudio software, and in particular frequency tables were created with the “table1” package. Barplots were created with the “ggplot2” function to visualize the descriptive results.

To summarize and obtain a unique measurement for each subject at WP1 and WP2, the mode was applied as a summary measure for the most consumed and preferred dish by each subject over time. This approach allowed identifying the most frequent category of dishes for each characteristic, such as the most common color, predominant odor, most frequent consistency, and preferred temperature.

It is important to emphasize that, given the nature of the study, no formal statistical tests were conducted. Given the descriptive nature of the analysis and the limited sample size, the focus was on using the mode to summarize the food preferences of individuals with ASD at WP1 and WP2. This approach provides an overview of common tendencies within the sample without the ability to make statistically generalizable inferences to the larger population.

## 3 Results

The main demographic, anthropometric and clinical characteristics of the 22 enrolled subjects were obtained from their medical records. The mean age of the overall population was 29.5 ± 7.1 years, and most study participants were males (72.7%, *n* = 16). ASD severity levels were distributed as follows: 72.7% (*n* = 16) of individuals with a diagnosis of third-level ASD severity, 22.7% (*n* = 5) with second-level ASD severity and 4.5% (*n* = 1) with first-degree ASD severity. According to BMI (16), the prevalence of overweight was 54.5% (*n* = 12) and obesity was 18.2% (*n* = 4); considering body fat distribution by means of waist circumference cut-off (17), the prevalence of abdominal obesity was 40.9% (*n* = 9).

### 3.1 Results related to the WP1

Regarding the acceptance of meals consumed by the subjects enrolled in the study, lunches took place at the *Sacra Famiglia Fondazione Onlus* in Cesano Boscone (MI). Table 1 presents the frequency of Consumption (mode measurement) and Sensory Characteristics of Food and Beverage Intakes registered at WP1. The description of the first course included: name of the dish, shape, possible addition of grated cheese, color, smell, texture, temperature. The description of the second course, bread/grissini/cracker, vegetable side and fruit/yogurt included: name of the dish, color, smell, texture, temperature. The description of the beverages included: name, number of glasses drunk, color, smell, temperature. Indeed, throughout the meal evaluation forms collected during lunchtime, the acceptance of meals was associated with the sensory characteristics of food (color, smell, texture and temperature).

**Table 1:** Frequency of consumption and sensory characteristics of food and beverage intakes at WP1.

First course		
		n (%)
Name	Risotto with zucchini and Prague; Pasta with pesto; Pasta with zucchini and saffron	10 (45.5)
	Pasta with tomato, mozzarella and basil; Pasta with tomato; Pizzaiola risotto	12 (54.5)
Pasta format	Fusilli	17 (77.3)
	Rice	5 (22.7)
Grated cheese	Yes	19 (86.4%)
	No	3 (13.6%)
Color	Red, white, and green	1 (4.5%)
	Red and white	21 (95.5%)
Smell	Faint	22 (100.0)
Texture	Soft	22 (100.0)
Temperature	Hot	22 (100.0)

Second course		
		n (%)
Name	Flounder gratin; Halibut; Trout burgers with herbs; Swordfish with salmoriglio; Cod and tomato medallions	8 (36.4%)
	Roast loin in green sauce; Chicken; Salami; Roast turkey with vegetables	14 (63.6%)
Color	Beige	21 (95.5%)
	Brown e green	1 (4.5%)
Smell	Faint	22 (100.0)
Texture	Soft	22 (100)
Temperature	Hot	22 (100)

Vegetables side dish		
		n (%)
Name	Tomatoes	5 (22.7%)
	Broccoli; Brussels sprouts	1 (4.5%)
	Green beans in aromatic oil; Zucchini	15 (68.2%)
	Carrots cooked with oil and onion; Parsley carrots; Carrots with oil	1 (4.5%)
Color	Green	22 (100)
Smell	Faint	22 (100)
Texture	Soft	22 (100)
Temperature	Hot	22 (100)

Bread/grissini/crackers		
		n (%)
Name	Bread	18 (81.8%)
	Whole flour bread	2 (9.1%)
	Crackers	2 (9.1%)
Color	Beige	20 (90.9%)
	Brown	2 (9.1%)
Smell	Faint	22 (100.0)
Texture	Soft	20 (90.9%)
	Chruncy	2 (9.1%)
Temperature	Room temperature	22 (100.0)

Fruits		
		n (%)
Name	Apricot with peel; Apricot without peel; Plum; Peach with peel; Peach without peel	12 (54.5%)
	Banana	3 (13.6%)
	Watermalon; Cantaloupe	7 (31.8%)
Color	Orange	6 (27.3%)
	Yellow	5 (22.7%)
	Red	11 (50.0%)
Smell	Faint	22 (100.0)
Temperature	Room temperature	21 (95.5%)
	Cold	1 (4.5%)
Mode of presentation	None	1 (4.5%)
	Whole fruit	7 (31.8%)
	Chopped fruit	14 (63.6%)

Beverages		
		n (%)
Name	Water	22 (100)
Number of glasses	1	9 (40.9%)
	2	7 (31.8%)
	3	6 (27.3%)
Color	Clear	22 (100.0)
Smell	Faint	22 (100.0)
Temperature	Room temperature	22 (100.0)

Shows the number (%) of subjects who consumed the most frequently recorded dishes and the sensory characteristics of them.

The description of the first course includes: name of the dish, shape, possible addition of grated cheese, color, smell, texture, temperature.

The description of the second course includes: name of the dish, color, smell, texture, temperature.

The description of bread/grissini/cracker includes: name, color, smell, texture, temperature.

The description of the vegetable side dish includes: name, color, smell, texture, temperature.

The description of fruit/yogurt includes: name, choice, color, smell, texture, temperature.

The description of the beverages includes: name, number of glasses drunk, color, smell, temperature.

With respect to the 12 surveys collected, the number of study participants consuming the most frequently recorded and administered dishes and their sensory characteristics is described in number and percentage.

The results of WP1 describe how the most frequently recorded meals have sensory characteristics in agreement with the scientific literature describing the individual food preferences of individuals with ASD in relation to color, smell, texture and temperature. The most recorded meals are the ones often found on menus and most frequently chosen and consumed by individuals.

The main characteristics that make a meal more accepted by individuals with ASD derived from the analysis of quantitative and qualitative forms of the standard menus and are summarized below:

- short pasta formats (such as bucatini, penne, orecchiette, farfalle)
- foods with low intensity colors such as white and beige;
- homogeneity of hues within the same dish;
- dishes containing ingredients of similar, non contrasting color tones;
- green vegetables are more accepted instead of the orange ones;
- soft or semi-soft textures, rejecting foods difficult to chew;
- muted odors and not pungent/strong, such as those of some fish species (e.g., mackerel);
- mild tastes, no to bitter and sour, reducing the use of strong taste spices, garlic and onion;

Summarizing what was observed in WP1 and the scientific literature on this topic, the authors produced a paper published in an indexed journal (18).

### 3.2 Results related to the WP2

The results observed at WP1 represented the starting point for the revision of targeted menus and the adaptation of collective catering menus responding to the nutritional and sensory needs of individuals with ASD.

With respect to energy requirements, the authors referred to the comparison made between energy intakes given by standard menus and energy requirements assessed by considering both ideal and actual weights of the enrolled subjects. Accordingly, the adapted menus provided about 800 kcal [35% of 2000 kcal corresponding to the daily requirement for an adult (19)], compared with the 960 kcal provided by the standard menus. To comply with a better distribution of macronutrients, first courses seasoned with vegetables or vegetable sauces, main courses based on legumes, fish, and white meat were preferred. The cheese portion was reduced from 70 g to 50 g, and side dishes with seasonal vegetables were included in place of potatoes or polenta, which was decided to be presented as a first course. Relative to the sensory aspect, long pasta shapes were replaced with short pasta shapes, monochromatic dishes were proposed (e.g., saffron risotto, pumpkin velouté, pea cream…), and dishes with soft tender textures and little intense colors and smells; furthermore, diced and peel-less fruits were preferred to facilitate chewing and soft-colored, and fruit salads were avoided to avoid mixing different colors and textures.

The acceptance of the targeted menus by the subjects enrolled in the study was evaluated and is presented in Table 2. The authors compared the score, calculated through the quantities of individual dishes consumed collected in the meal evaluation forms, related to WP1 (standard menus) with the score calculated according to the food consumption in WP2 (targeted menus).

**Table 2:** Meals acceptance recorded in WP1 and WP2.

	WP1 (*n* = 22)	WP2 (*n* = 22)
First course		
Media ± SD	0.657 ± 0.369	0.673 ± 0.397
Mediana (min;max)	0.826 (0;1)	0.913 (0;1)
Second course		
Media ± SD	0.663 ± 0.292	0.698 ± 0.335
Mediana (min;max)	0.667 (0;1)	0.784 (0;1)
Bread/grissini/crackers		
Media ± SD	0.748 ± 0.391	0.742 ± 0.365
Mediana (min;max)	1.00 (0;1)	0.917 (0;1)
Vegetable side dish		
Media ± SD	0.515 ± 0.367	0.573 ± 0.418
Mediana (min;max)	0.547 (0;1)	0.625 (0;1)
Fruits		
Media ± SD	0.654 ± 0.404	0.642 ± 0.382
Mediana (min;max)	0.818 (0;1)	0.833 (0;1)
Yogurt		
Media ± SD	0.063 ± 0.213	0.052 ± 0.14
Mediana (min;max)	0 (0;1)	0 (0;0.66)
Beverages		
Media ± SD	0.992 ± 0.035	0.989 ± 0.029
Mediana (min;max)	1.00 (0.88;1)	1.00 (0.91;1)

Shows the comparison between semiquantitative consumption of the different meals, including first course, second course, bread/grissini/cracker, vegetables, fruits/yogurt, beverages. Total score of WP1 and WP2 expressed in both mean ± SD and median (min;max). WP, Work Package.

The results describe an increase in the score for all the meal dishes (first course, second course, vegetable side dish, fruit) at WP2 compared to WP1. A slight score deflection is observed for bread/grissini/cracker consumption, while yogurt is not consumed at either WP1 or WP2, as individuals prefer fruit consumption to it. The beverages consumption was adequate since WP1 and did not change with the adapted menus.

## 4 Discussion

The FOOD-AUT project finds an important level of uniqueness. Several studies aimed at improving food selectivity focus mainly on implementing psychotherapeutic approaches such as treatments with differential reinforcement of alternative behaviors-one (20, 21). However, to date, no studies have been published associating the sensory and nutritional needs of individuals with ASD and the collective catering service.

Notably, most research projects with this objective target a population of children as scientific literature describes how acting at an early age has a greater chance of improvement (4, 8). Therefore, food selectivity in older patients could be a challenging issue. FOOD-AUT, by targeting an adult population, aimed to provide support to those individuals who may have established certain attitudes toward food (4, 8).

The qualitative improvements observed in FOOD-AUT project showed how a targeted action responding to the sensory needs of this population, led to an improvement in food consumption, even in adults who have more consolidated eating patterns and consequently have more difficulty accepting changes.

Notably, comparing the results of WP1 with the results of WP2, the authors observed a higher adherence and consumption in terms of quantity of meals at WP2. For both first course, second course, vegetables and fruits the median score was higher at WP2 than at WP1.

It is important to emphasize how an increase in the consumption of fruits and vegetables can lead to a higher intake of vitamins such as A, C, folate and minerals, particularly iron and zinc of which individuals with ASD and food selectivity are often deficient (22–25). In addition, a increased consumption of main courses such as fish can lead to increased intake of calcium, vitamin D and omega-3, also often deficient in this vulnerable population (26, 27). For regard to beverages, water consumption was already adequate at WP1, and there was no change was recorded at WP2. The reduction in bread consumption in WP2 can, on the other hand, be justified by the fact that since consumption of first courses and side dishes, subjects no longer had a need to consume a large portion of bread to satiate themselves.

These results indicate how menu adaptation incentivizes food consumption by people with ASD, consequently decreasing food waste and improving their food choices.

In conclusion, given the noble goal of the project and the encouraging results, it is hoped that the FOOD-AUT project will be continued, testing the effectiveness of the nutritional claims and menus produced, on a larger sample size and different age groups, raising awareness of the need to draft dietary guidelines at a national level.

## 5 Strengths and limitations

The current pilot study had a relatively small sample size and lacked a control group. It should also be noted that the small sample size may limit the representativeness of the results and the generalizability of the conclusions. Additionally, the high prevalence of males in the study population, while consistent with the higher prevalence of males in ASD, cannot be considered a representative reflection of the broader population under investigation. Furthermore, the use of the mode as a summary measure for categorical variables may simplify the information contained in the data. Future controlled studies with wider samples are needed to investigate these preliminary findings further. Nevertheless, the great uniqueness of the study with respect to the topic investigated should be underlined. In fact, no previous study has focused on evaluating the meals provided by collective catering in individuals with ASD. Moreover, the results of the present pilot study on individuals with ASD are promising with a greater meal acceptance and lower food waste after administration of the adapted menus.

## Data availability statement

The raw data supporting the conclusions of this article will be made available by the authors, without undue reservation.

## Ethics statement

The studies involving humans were approved by Ethics Committee of the University of Pavia (Comitato Etico del Dipartimento di Scienze del Sistema Nervoso e del Comportamento – sez. di Psicologia), project. n. 114/22. The studies were conducted in accordance with the local legislation and institutional requirements. Written informed consent for participation in this study was provided by the participants’ legal guardians/next of kin. Written informed consent was obtained from the individual(s) for the publication of any potentially identifiable images or data included in this article.

## Author contributions

MC: Writing – original draft, Writing – review & editing. SS: Writing – original draft. CB: Writing – original draft. SB: Writing – original draft. GF: Writing – original draft. AL: Writing – original draft. LV: Writing – original draft. RC: Writing – original draft. CT: Writing – original draft. HC: Supervision, Writing – original draft, Writing – review & editing.
